# Altered intrinsic excitability of hippocampal CA1 pyramidal neurons in aged PDAPP mice

**DOI:** 10.3389/fncel.2015.00372

**Published:** 2015-10-14

**Authors:** Francesco Tamagnini, Janet Novelia, Talitha L. Kerrigan, Jon T. Brown, Krasimira Tsaneva-Atanasova, Andrew D. Randall

**Affiliations:** ^1^Medical School, University of ExeterExeter, UK; ^2^School of Physiology and Pharmacology, University of BristolBristol, UK; ^3^Department of Mathematics, College of Engineering, Mathematics and Physical Sciences, University of ExeterExeter, UK

**Keywords:** PDAPP, ageing, hyperexcitability, hypoexcitability, amyloidopathy, hippocampus, Alzheimer’s disease

## Abstract

Amyloidopathy involves the accumulation of insoluble amyloid β (Aβ) species in the brain’s parenchyma and is a key histopathological hallmark of Alzheimer’s disease (AD). Work on transgenic mice that overexpress Aβ suggests that elevated Aβ levels in the brain are associated with aberrant epileptiform activity and increased intrinsic excitability (IE) of CA1 hippocampal neurons. In this study we examined if similar changes could be observed in hippocampal CA1 pyramidal neurons from aged PDAPP mice (20–23 month old, *Indiana* mutation: V717F on APP gene) compared to their age-matched wild-type littermate controls. Whole-cell current clamp recordings revealed that sub-threshold intrinsic properties, such as input resistance, resting membrane potential and hyperpolarization activated “sag” were unaffected, but capacitance was significantly decreased in the transgenic animals. No differences between genotypes were observed in the overall number of action potentials (AP) elicited by 500 ms supra-threshold current stimuli. PDAPP neurons, however, exhibited higher instantaneous firing frequencies after accommodation in response to high intensity current injections. The AP waveform was narrower and shorter in amplitude in PDAPP mice: these changes, according to our *in silico* model of a CA1/3 pyramidal neuron, depended on the respective increase and reduction of K^+^ and Na^+^ voltage-gated channels maximal conductances. Finally, the after-hyperpolarization, seen after the first AP evoked by a +300 pA current injection and after 50 Hz AP bursts, was more pronounced in PDAPP mice. These data show that Aβ-overexpression in aged mice altered the capacitance, the neuronal firing and the AP waveform of CA1 pyramidal neurons. Some of these findings are consistent with previous work on younger PDAPP; they also show important differences that can be potentially ascribed to the interaction between amyloidopathy and ageing. Such a change of IE properties over time underlies that the increased incidence of seizure observed in AD patients might rely on different mechanistic pathways during progression of the disease.

## Introduction

In the last 10–15 years, amyloid beta (Aβ) overexpressing transgenic mouse models have become a widely utilized tool for the exploration of the pathogenesis and the molecular mechanisms underlying amyloidopathies, neurodegenerative diseases characterized by the accumulation of Aβ in various forms, including extracellular plaques. Alzheimer’s disease (AD) is a neurodegenerative disease characterized by both amyloidopathy (Hardy and Higgins, 1992; Hardy, 2009; Pimplikar, 2009) and tauopathy (i.e., the intracellular accumulation of hyper-phosphorylated tau neurofibrillary tangles) (Selkoe, 2001). Amyloidopathy results from the sequential cleavage of the amyloid precursor protein (APP) by β- and γ-secretase enzyme complexes, respectively (Citron, 2004; Thinakaran and Koo, 2008). So far, research has mainly focused on the mechanisms underlying synaptic deficits caused by amyloidopathy, since alterations in synaptic transmission and network function are widely regarded as the main cellular correlate for early cognitive decline, observed in both AD patients and Aβ-overexpressing transgenic mice (Puzzo et al., 2005; Shankar et al., 2008; Randall et al., 2010; Witton et al., 2010; Sheng et al., 2012; Tamagnini et al., 2012; Crimins et al., 2013; Ripoli et al., 2013). Much of this work has focused on hippocampus and/or cerebral cortex, CNS structures which are mainly involved in the higher order processing of sensory information and resultant cognitive abilities, aspects of the CNS that are mainly affected in AD. Hippocampal function and structure, and in particular its CA1 subfield, have been massively studied in the last 30 years, for their central role in episodic memory encoding and spatial processing (reviewed in Bird and Burgess, 2008); particular attention has been paid to hippocampal changes in physiological aging and dementia and how such modifications correlate and are causally related to memory and cognitive decline (Moodley and Chan, 2014; Gray and Barnes, 2015). One of the main clinical outcomes of AD, other than memory impairment and cognitive decline, is seizure: in fact, AD patients suffer from a higher incidence of seizure (Amatniek et al., 2006). In relation to this clinical observation, recent work was conducted in our and other labs, focusing on the characterization of the disruption of hippocampal and cortical neuronal synchronized activity in models of AD (Palop et al., 2007; Minkeviciene et al., 2009; Putcha et al., 2011; Verret, 2012; Corbett et al., 2013; Born et al., 2014; Davis et al., 2014); further research efforts were also spent on the characterization of the intrinsic excitability (IE) alterations of neuronal properties in mouse models of amyloidopathy, such as PSAPP (Brown et al., 2011), PDAPP (Kerrigan et al., 2014), CRND8 (Wykes et al., 2012), 5xFAD (Kaczorowski et al., 2011), APPSwe/PS1dE9 (Kellner et al., 2014), soluble oligomers of Aβ 1–42 (Scala et al., 2015; Tamagnini et al., 2015), 3xTg APPSwe/PS1/P301L (Scala et al., 2015), Tg2576 (Brown et al., 2011; Nenov et al., 2015) and APP23xPS45 (Busche et al., 2012).

Most reported IE changes associated with amyloidopathy point towards hyper-excitability, such as the increased action potential (AP) firing rate, which has been shown to be increased in several Aβ overexpressing transgenic mouse models (Brown et al., 2011; Wykes et al., 2012; Kerrigan et al., 2014; Scala et al., 2015). Our previous work conducted on PSAPP mice (Swedish mutation, K670N, M671L on APP + M146L on PS1), showed that hyper-excitability, expressed as higher firing rate and narrower, smaller (in amplitude) spikes, correlates and it is likely causally linked to the ~50% reduction of Na^+^ voltage-gated currents observed in outside-out nucleated macropatches, excised from the cell body of CA1 pyramidal neurons of PSAPP vs. wild-type (WT) littermate controls (Brown et al., 2011). Reduced AP width and increased firing rate have also been observed in 8–9 month old PDAPP mice (Kerrigan et al., 2014). Other work, conducted on CRND8 mice (Wykes et al., 2012), partially confirmed what we previously observed in PSAPP and PDAPP mice: a narrower AP width was seen, which in these animals was suggested to be related to an increased expression of Kv3.1 channels. These results collectively suggest that amyloidopathy can be associated to increased excitability in hippocampal cells due to alterations of the biophysical properties of voltage-gated channels involved in the AP generation. In another instance, extracellular Aβ 1–42 oligomers (500 nM) bath application to hippocampal slices from 1 month old WT mice, did not affect the firing rate but reduced the after-hyperpolarization (AHP) and hyperpolarized the AP threshold (Tamagnini et al., 2015). Furthermore, intracellular application of Aβ oligomers (at a lower concentration, 200 nM) in isolated primary neurons cultured from P0–2 pups, resulted in higher firing rate but wider AP width (Scala et al., 2015). Conversely, other bodies of data suggest that amyloidopathy relates to decreased IE; for example, hippocampal neurons from 8 month old 5xFAD mice showed a larger AHP compared to their controls, whereas no difference was observed at 2 month of age (Kaczorowski et al., 2011). Finally, physiological aging, not associated with amyloidopathy, is linked to a reduced IE in terms of slower firing rate (Randall et al., 2012) and increased AHP (Disterhoft and Oh, 2007).

These data can be collectively summarized thus: (1) amyloidopathy and physiological ageing are associated with increased and decreased IE of hippocampal neurons, respectively; (2) the effects of toxic amyloid species (such as any other toxic agent) on hippocampal neurons differ between an acute (Scala et al., 2015; Tamagnini et al., 2015) and chronic exposure (Brown et al., 2011; Wykes et al., 2012; Kerrigan et al., 2014); (3) ageing and amyloidopathy can interact resulting in alterations towards both increased and decreased IE, depending on the IE property considered (Kaczorowski et al., 2011; Scala et al., 2015).

Combined experimental and modeling studies of neuronal excitability often provided an important insight into the mechanisms regulating specific brain functions. The benefit of using mathematical and computational models to characterize neuronal behavior is that they build an overall representation of a system that can incorporate information from different levels of description e.g., molecular and/or cellular processes. Mathematical models are beginning to be applied in studies of neurodegenerative diseases, such as in modeling full Alzheimer disease pathology (Puri and Liwu, 2010) as well as, and of particular relevance to the present study, Aβ related excitability changes (Nowacki et al., 2011; Culmone and Migliore, 2012; Zou et al., 2012).

Following our previous study on younger (8–9 month old) PDAPP mice (Kerrigan et al., 2014), the aim of the present work is to test the effect of both genotype and age on the IE properties of hippocampal CA1 pyramidal neurons (CA1-PC) in older (20–23 month old) PDAPP mice vs. age-matched WT littermate controls; subsequently, we employed our unified Hodgkin-Huxley-type *in silico* model of CA1/3 pyramidal neuron (Nowacki et al., 2011) in order to verify the potential role of alterations in Na^+^ and/or K^+^ voltage-gated channel biophysical properties as a mechanistic correlate of the AP waveform property changes observed in the current study.

## Materials and Methods

### Experimental Animals

Male PDAPP (*n* = 9) transgenic (Tg) mice and age-matched WT littermate controls (*n* = 9) were used. This mouse line expresses a mutated form of the human APP gene carrying the V717F mutation (*Indiana*). As previously described (Games et al., 1995) the expression of this transgene in the PDAPP model is controlled by the platelet-derived growth factor (PDGF) β-chain promoter, resulting in progressive age-dependent extracellular accumulation of Aβ peptides. The present study was performed in 20–23 month old animals and the experiments were designed in order to compare PDAPP mice and age-matched littermate WT controls. All animals were supplied by Eli Lilly Pharmaceuticals (UK) as part of the European Union’s Pharmacog initiative. Animals were housed with free access to food and water *ad libitum* and kept on a standard 12:12 light/dark cycle. This study was carried out in accordance with UK Home Office Guidelines and the University of Exeter Animal Welfare Ethical Review Board. The protocol was approved by the University of Exeter Animal Welfare Ethical Review Board.

### Preparation of Brain Slices

Preparation of horizontal ventral hippocampal slices was performed as previously described (Brown and Randall, 2009; Brown et al., 2011; Tamagnini et al., 2015). In brief, mice were sacrificed by cervical dislocation. The brain was rapidly removed and transferred to an ice cold (~4°C), sucrose-based slicing solution comprising (in mM): sucrose, 189; D-glucose, 10; NaHCO_3_, 26; KCl, 3; MgSO_4_, 5; CaCl_2_, 0.1; NaH_2_PO_4_, 1.25; the solution was continuously bubbled with carbogen (95% O_2_, 5% CO_2_) gas mixture. The cerebellum, frontal and dorsal parts were removed with single scalpel cuts. The brain was then mounted on a metal plate on the dorsal side (ventral side up) and 300 μm thick horizontal sections were prepared using a Leica VT1200 vibratome. After sectioning, slices were submerged in a storage vessel which contained artificial cerebrospinal fluid (aCSF) consisting of (in mM): NaCl, 124; KCl, 3; NaHCO_3_, 24; CaCl_2_, 2; NaH_2_PO_4_, 1.25; MgSO_4_, 1; D-glucose, 10; the aCSF was equilibrated with carbogen gas mixture. The slices were gradually heated to ~32–34°C for 30 min, after which they were stored at room temperature (25°C) for 60–90 min. Each slice was then transferred to the submersion style recording chamber and was continuously perfused with carbogen bubbled aCSF, at a constant flow-rate (1–2 ml/min^−1^) and kept at 33 ± 1°C. Pyramidal neurons were visually identified in the stratum pyramidale of the CA1 subfield of the hippocampus (CA1-PC) using infra-red DIC microscopy. Pipettes were fabricated from borosilicate glass and were fire polished such that their resistance was 2.5–4.5 MΩ when filled with pipette solution. The pipette solution consisted of (in mM): K-gluconate, 145; NaCl, 5; HEPES free acid, 10; EGTA, 0.2; Na-GTP, 0.3; Mg-ATP, 4; pH 7.3, 280–290 mOsm. After the formation of the giga-seal and entering whole-cell configuration in voltage-clamp (VC) mode, the amplifier was switched to bridge-mode current-clamp (CC). The pairing of the pipette solution and the aCSF generates a liquid junction potential error of 15 mV; this was arithmetically corrected in all CC recorded data-sets. All recordings were performed using a MultiClamp 700B amplifier (Molecular Devices, Union City, CA, USA). Recordings were lowpass filtered (5–10 kHz) and subsequently digitized (sampling frequency: 100 kHz) with a Digidata 1440 (Molecular Devices, Union City, CA, USA), visualized and stored on a personal computer using pClamp10 electrophysiology software.

### *In Vitro* Electrophysiology Protocols and Data Analysis

**Figure 1:**
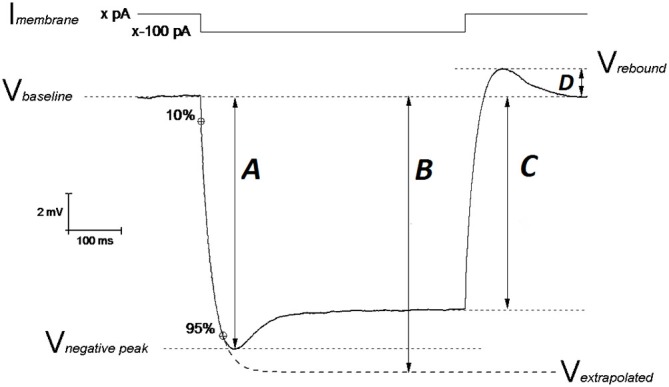
**Schematic representation of V_m_ hyperpolarization induced by a 500 ms negative current step: measuring the passive membrane properties**. The IE properties of CA1-PCs were assessed by performing whole cell current-clamp (CC) recordings. In order to avoid biases arising from cell-to-cell variability in resting membrane potential (RMP), each IE property (except RMP) was measured at an unstimulated V_m_ of −80 mV (V_baseline_): this value was kept fixed with a constant current (I_membrane_; xpA) injection. A 500 ms long, −100 pA current step was injected in order to assess the neuronal membrane passive properties. The input resistance was measured at the steady state of the V_m_ deflection (arrow C) or at the infinite point (arrow B) in the first exponential fit extrapolated between 10–95% of the negative peak (arrow A), which approximates the V_m_ deflection induced by the same hyperpolarizing current in the absence of I_h_ determined “SAG”. The exponential fit was also used to calculate the membrane time constant τ. The SAG was calculated both vs. the negative peak ((A–C)/A)) and vs. the V_m_ extrapolated at the infinite time of the exponential fit ((B–C)/B)). The same mechanistic reasons generating SAG, are responsible for the rebound observed upon repolarization (arrow D). Modified from Tamagnini et al. (2015).

In this study we gathered electrophysiological recordings from 18 slices from 9 PDAPP Tg and 14 slices from 9 WT littermate control brains: each day 1 animal was sacrificed and the entire study was distributed over a period of 11 weeks. Analysis of CC recordings, including AP waveform analysis, was carried out with custom-written Matlab routines, as previously described (Tamagnini et al., 2015). Briefly, the resting membrane potential (RMP) was measured at the beginning of the recording. After assessing RMP, all other CC recordings were carried out at set pre-stimulus membrane potential (V_m_) of −80 mV. This was established using an appropriate amount of constantly injected bias current (x in Figure 1). Membrane input resistance (R_in_) was analyzed in 3 different ways: (1) R_in_ assessed independently of the “sag”-producing I_h_ activation, that occurs during hyperpolarization current steps (R_in-exp_). It was calculated according to Ohm’s law (V = IR) from the amplitude of the extrapolation at an infinite time of a single exponential curve fitted to the membrane charging response generated by a −100 pA current injection (Figure 1, arrow B). The fit has been made between 10% and 95% of peak amplitude (Figure 1, arrow A). This exponential fit has also been used to assess the membrane time constant (τ) and to measure the sag (see below); (2) R_in_ was calculated according to Ohm’s law by measuring the steady-state (post-sag) V_m_ deflection generated by −100 pA square current (R_in-ss_); this measure includes the contribution from additional I_h_ activation (Figure 1, arrow C). Finally, (3) R_in_ was also measured as the reciprocal slope of conductance in the linear regression of steady state voltage responses elicited by a series of low amplitude (−50 to +30 pA), 500 ms square current steps (Figure 3A).

Hyperpolarization-activated sag is caused by the depolarizing current (I_h_) generated by the opening of hyperpolarization-cyclic-nucleotide-activated channels (HCN) and it has been measured in two ways. The first measurement (sag_sub_) simply expresses the difference between the negative peak (Figure 1, arrow A) and steady state hyperpolarizations produced by a 500 ms, −100 pA current injection as a percentage of the peak hyperpolarization, i.e., 100 * (A–C)/A (in Figure 1). The second measure of sag (sag_fit_) measures the decay in response relative to the amplitude of the infinite time extrapolation used to determine R_in-exp_ i.e., 100 * (B−C)/B (in Figure 1). For the same mechanistic reasons, a rebound was observed upon repolarization and measured as the difference V_rebound_ − V_baseline_ (Figure 1, arrow D). Capacitance was calculated as the ratio between the τ (tau; ms) and R_in-exp_. All passive properties were statistically compared between genotypes using unpaired Student’s *t*-tests.

In order to asses sub-threshold membrane resonance properties, standard “ZAP” protocols were used as previously described (Hu et al., 2002; Tamagnini et al., 2015). Briefly, the impedance profile of CA1-PCs was calculated as the ratio between the Fast-Fourier transform of the voltage response (V(fft)) and current injection (I(fft)): Z = V(fft)/I(fft); the impedance vs. frequency profile was then smoothed with a moving average function with a span of 35 data-points. In order to compare the resonance profile between genotypes, cell-to-cell analysis was performed and the following properties quantified: the frequency of maximal impedance (Peak frequency, Hz), the maximal impedance (Z max, MΩ), and the quality factor of the resonator (Q). Unpaired Student’s *t*-tests were used to test the effect of genotype on resonance properties.

Depolarizing 500 ms square current injections with amplitude varying between 50 and 300 pA, were used to elicit AP firing. The total number of APs fired was plotted vs. the current intensity injection for each genotype. In addition, the instantaneous frequency (IF) of firing was also analyzed, as the frequency between two consecutive spikes and plotted against the interspike interval (ISI), in order to measure burstiness and frequency of firing after accommodation, between genotypes. The firing properties were examined between genotype groups using two-way analysis of variance (ANOVA). In order to describe individual AP waveforms, the first spike fired by a 300 pA current injection was analyzed. AP threshold was determined from phase plots as the voltage at which dV/dt surpassed the value of 15 V·s^−1^ (Naundorf et al., 2006). Spike width was measured at −15 mV which is approximately halfway between threshold (~−60 mV) and AP peak (~+30 mV). The AP maximal rate of rise (RoR) was also measured. In addition, the AHP was measured during firing, as the difference in mV between threshold and the minimum peak after the first AP evoked by a 300 pA square current injection. The AP properties were statistically compared between groups using unpaired Student’s *t*-tests.

In order to better isolate possible differences in post-spike events such as spike after-depolarization (ADP) or AHP, single APs were evoked using single, brief (2 ms) and strong (2 nA) current injections. Unpaired Student’s *t*-tests were used to compare the ADP and AHP elicited by a single spike.

To further investigate the effect of genotype on AHP, trains of 5, 10, 15, 20 or 25 square current pulses (2 ms, 2 nA) were delivered at a frequency of 50 Hz, such that each pulse elicited a single AP; the medium AHP amplitude (mAHP) was measured as the more hyperpolarized point comprised between the end of the train (when the V_m_, during the last AP descending phase, goes back to 1 mV from the baseline value) and the following 500 ms. Two-way ANOVA was used to statistically assess the effect of genotype on the mAHP elicited by 50 Hz spike trains.

For each measure, the *n* reported in the “Result” section and relative figures, represents the number of cells we recorded from.

### Modeling

In order to model the effect of genotype on the hippocampal CA1 pyramidal neurons AP peak and width in older (20–23 month old) PDAPP mice vs. age-matched WT littermate controls, we used our CA1/CA3 pyramidal neuron model (Nowacki et al., 2011). The model was fit to reproduce the WT average AP waveform data from the present study (the first AP elicited by a 300 pA, 500 ms square wave current) and then systematically investigated by changing the Na^+^ and K^+^ voltage-gated conductance, in order to account for the PDAPP experimental observations. Model simulations were performed using Matlab.

## Results

**Figure 2:**
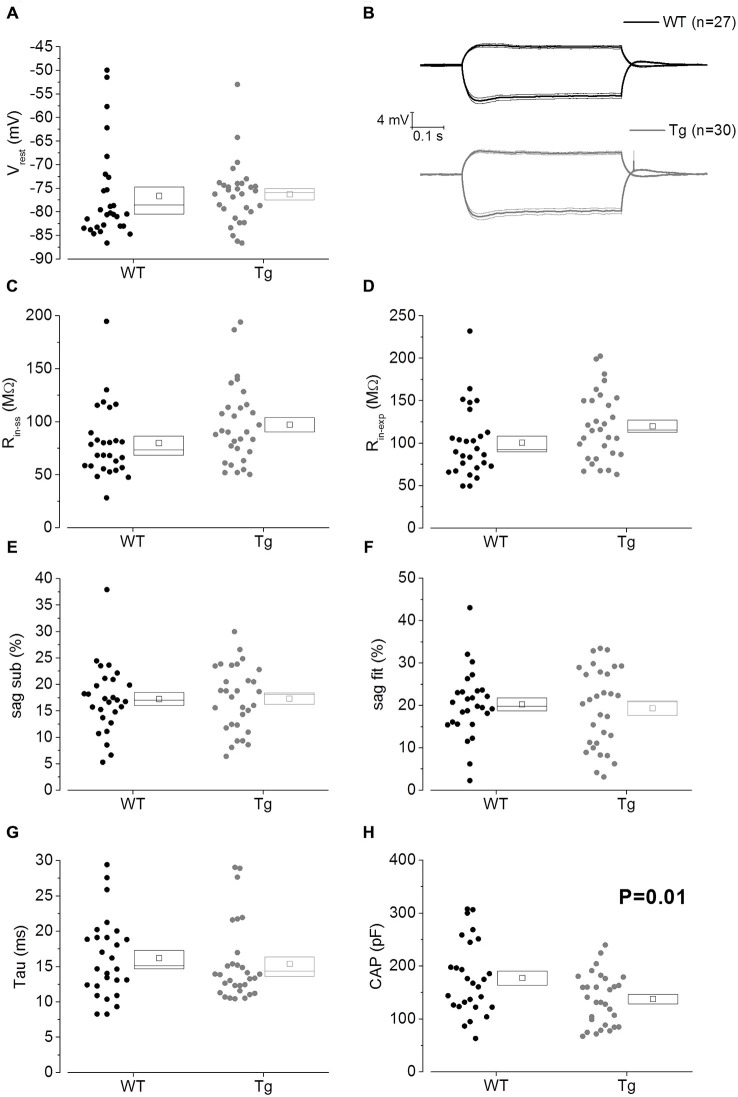
**20–23 month old PDAPP mice have a lower capacitance but do not show any other difference in passive membrane properties. (A)** Scatter plot/box showing cell-to-cell zero current RMPs (in figure: V_rest_) recorded from PDAPP and WT CA1-PCs: no significant differences were observed between genotypes. In this and other similar plots, the scattered symbols on the left represent data coming from single cell analysis, while the box to the right plots the mean (central symbol), the ± SEM (lower and upper boundary of the box) and the median. **(B)** Voltage responses of WT (black lines) and PDAPP (gray lines) hippocampal CA1-PCs in response to +50 pA and −100 pA, 500 ms current steps; thick lines are the averaged traces, thin lines represent the SEM. The negative responses were used to measure sub-threshold membrane properties derived from −100 pA stimuli applied at a fixed membrane potential of −80 mV, as represented in scatter plots-box **(C–H)**: no effect of genotype was observed on most of these properties **(C–G)** but a very strong effect was observed on capacitance **(H)** which was significantly reduced in PDAPP mice. See Table 1 for average ± SEM and *p* values.

**Figure 3:**
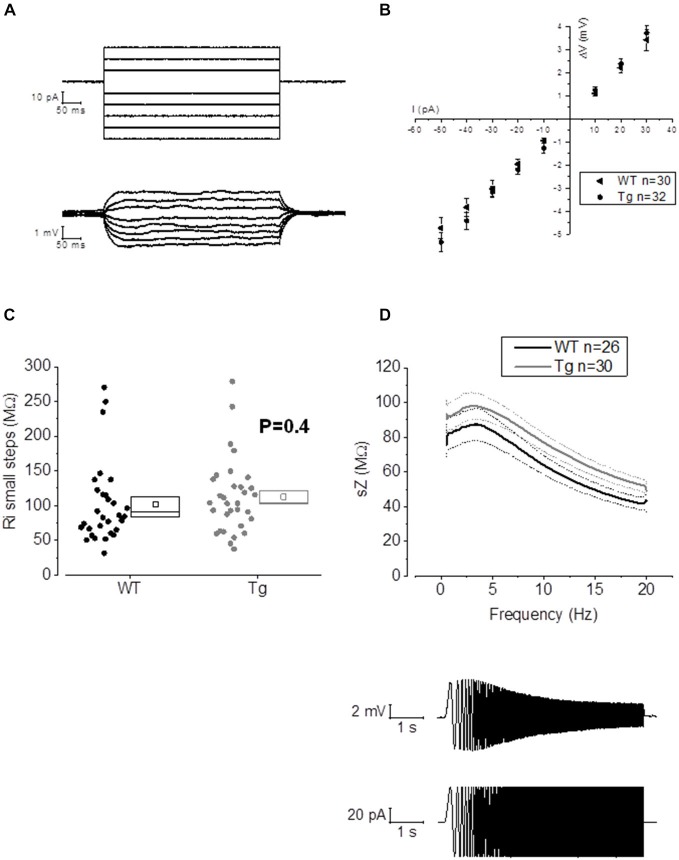
**20–23 month old PDAPP mice do not show alterations of membrane input resistance and impedance profile in CA1-PCs**. **(A)** Examples of voltage responses evoked in a CA1-PC: these were elicited by a series of 500 ms current stimuli varying in amplitude between −50 and +30 pA. **(B)** Pooled data of R_in_ recordings, including the example shown in **(A)**. No difference was observed between genotypes in R_in_ measured with this method, which corresponds to the inverse of the slope of the straight line interpolated, for each cell, in an IV plot (Ohm’s law: V = IR). **(C)** Scatter/box plot showing cell-to-cell analysis of the R_in_ calculated with the small steps method. **(D)** Top panel: smoothed mean impedance (sZ) plotted vs. stimulus frequency for WT (black) and PDAPP (gray) CA1-PCs. The central line (thicker) represents the mean values while the bounds of 1 SEM are represented as dashed lines. Bottom panel: example of a CA1-PC V_m_ resonating in response to a sinusoidal current of increasing frequency. The quality factor of the resonator, Q, is calculated as the ratio between the Z at peak frequency and Z at the frequency of 1 Hz (Q = Z_*peak*_/Z_1 *Hz*_). The impedance Z(Ω) is measured as Z = V(fft)/I(fft). The genotype showed no effect on the impedance profile of hippocampal CA1-PCs from PDAPP mice. See Table 2 for average ± SEM and *p* values.

The main aim of this study was to assess the effect of genotype on CA1-PC IE in transgenic aged Aβ overexpressing PDAPP mice. As shown in Figure 2A, no significant effect was observed on the RMP. Figure 2B shows the group average ± SEM voltage traces in response to −100 pA and +50 pA square current stimuli applied at a pre-stimulus potential fixed at −80 mV. It is readily apparent that responses in the two genotypes were very similar. In fact, cell-to-cell analysis of most of the sub-threshold membrane properties performed on voltage deflections in response to a −100 pA square current stimulus, showed no effect of genotype (Figures 2C–G). Membrane resistance, measured as R_in-ss_ (Figure 2C; *p* = 0.07) and R_in-exp_ (Figure 2D; *p* = 0.08) was unaltered; sag, measured as sag_sub_ (Figure 2E; *p* = 0.96) and sag_fit_ (Figure 2F; *p* = 0.69), was unaltered as well. As expected, the sag-related depolarizing rebound potential was not significantly different between PDAPP mice and their controls (data not shown; *p* = 0.33). The time constant (τ) of the V_m_ exponential decay in response to a −100 pA hyperpolarizing current injection was not significantly different between genotypes (Figure 2G; *p* = 0.57). Interestingly, the calculated membrane capacitance was significantly lower in PDAPP mice (Figure 2H). The lack of effect of the genotype on R_in_ was further confirmed when calculated as the reciprocal of the slope of the straight line function interpolating the steady-state voltage deflections plotted vs. the current step intensities (Figures 3A–C; *p* = 0.4). Table 1 summarizes the findings of the sub-threshold passive properties of PDAPP vs. WT CA1-PCs (average ± SEM and *p* values).

**Table 1:** **Passive membrane properties of CA1-PCs in hippocampal slices from 20 to 23 month old PDAPP mice and age-matched WT littermate controls**.

	WT *n* = 27		PDAPP *n* = 30	
Property	Average	SEM	Average	SEM	*p*
RMP (mV)	−76.7 (*n* = 28)	1.9	−76.3	1.2	0.88
Rin-ss (MΩ)	79.8	6.5	97.1	6.7	0.07
Rin-exp (MΩ)	100.2	8.0	119.9	7.3	0.08
sag_sub (%)	17.2	1.2	17.3	1.1	0.96
sag_fit (%)	20.2	1.5	19.3	1.7	0.69
Rebound (mV)	1.6	0.1	4.2	2.5	0.33
tau (ms)	16.2	1.1	15.4	1.0	0.57
Capacitance (pF)	177.2	13.4	137.4	8.8	0.01

The activation/deactivation gating properties of channels active around the RMP, such as voltage-gated K^+^ channels 7 (Kv7) and HCN channels, generating I_M_ and I_h_ respectively, are responsible for the sub-threshold resonance properties of neurons (see “Materials and Methods” section for details; Hu et al., 2002). Cell-to-cell analysis of peak resonant frequency (Peak frequency, Hz), peak impedance (Z, MΩ) and the quality factor of the resonator (Q) did not reveal a significant effect of genotype on resonance properties in CA1-PCs (Figure 3D). For a summary of the means ± SEM of CA1-PCs sub-threshold resonance properties in PDAPP vs. controls see Table 2.

**Table 2:** **Resonance properties of CA1-PCs in hippocampal slices from 20 to 23 month old PDAPP mice and age-matched WT littermate controls**.

	WT *n* = 26		PDAPP *n* = 30	
Property	Average	SEM	Average	SEM	*p*
Peak frequency (Hz)	3.73	0.37	3.77	0.28	0.93
Q	1.11	0.02	1.11	0.01	0.86
Peak Z (MΩ)	90.34	9.45	100.99	7.90	0.39

The lack of alteration of sub-threshold passive and resonance membrane properties in PDAPP mice is in accordance with previous work on CA1-PCs in younger PDAPP mice (Kerrigan et al., 2014) and other Aβ overexpressing tg mouse lines (Brown et al., 2011; Wykes et al., 2012), as well as normal hippocampal slices pre-treated with soluble Aβ oligomers (Tamagnini et al., 2015); however, the decrease in capacitance observed in the current study is a novel finding.

**Figure 4:**
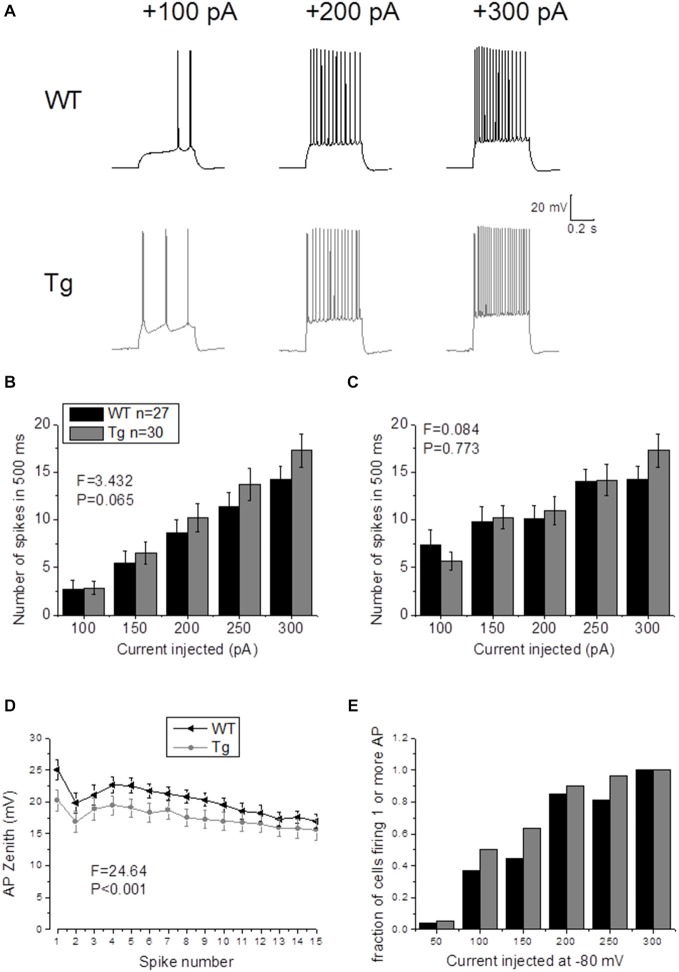
**20–23 month old PDAPP mice do not show altered firing frequency properties of CA1-PCs but are characterized by hyperpolarized action potential (AP) zeniths during tonic firing. (A)** CA1-PCs from either PDAPP or WT CA1-PCs fired APs in response to supra-threshold, 500 ms current steps. **(B)** The genotype showed no effect on the average number of APs fired by CA1-PCs in response to 50–300 pA, 500 ms current steps, including or excluding **(C)** the cells that did not fire at least 1 AP. **(D)** On the other hand, CA1-PCs from PDAPP mice showed an overall reduction of the AP peak measured within the first 15 APs fired by the cells in response to a 300 pA, 500 ms current step. **(E)** Genotype showed a trend to increase the fraction of firing cells, for each applied current intensity tested.

In order to study supra-threshold CA1-PCs properties in PDAPP mice, 500 ms depolarizing current stimuli were applied and AP firing properties were analyzed. Figure 4A shows the firing profile of an example cell for each respective genotype at 3 of the different current intensities tested. The mean number of spikes fired for each current amplitude (including non-firing sweeps), was not significantly higher in PDAPP CA1-PCs compared to WT littermate controls (Two-way ANOVA: Source of variation: Genotype; DF = 1; *F* = 3.432, *p* = 0.065; Figure 4B); however, a trend in the average towards a higher number of APs in PDAPP mice was noticed. Similarly, no significant difference (Two-way ANOVA: Source of variation: Genotype; DF = 1; *F* = 0.084, *p* = 0.773) was observed between genotypes in the average number of spikes fired at each current intensity tested when non-firing cells were excluded (Figure 4C). The time course analysis of the AP zenith measured between genotypes within the first 15 fired spikes, revealed that PDAPP CA1-PCs fire APs with less depolarized peaks (Two-way ANOVA: Source of variation: Genotype; DF = 1; *F* = 24.62, *p* < 0.001; Figure 4D). Finally, PDAPP mice showed a trend towards higher fractions of firing cells at the tested current intensities compared to littermate controls (Figure 4E).

**Figure 5:**
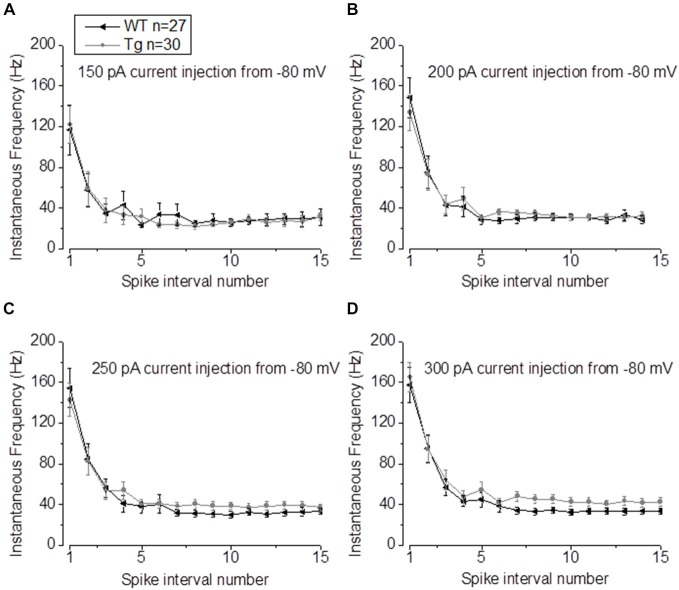
**20–23 month old PDAPP mice show higher plateau instantaneous firing in CA1-PCs only at intense current injections. (A–C)** Injection of 150–200–250 pA, 500 ms current steps did not alter the IF profile of CA-PCs from 20 to 23 month old PDAPP mice. **(D)** However, at a higher current injection (300 pA), the IF of CA1-PCs in the same mice stabilized at a higher plateau value compared to age-matched WT littermate controls.

In our previous studies, we observed that the average firing was increased in 9–10 month old PDAPP mice (Kerrigan et al., 2014) and in PSAPP mice (Brown et al., 2011). This effect was more evident if the IF was considered, because of the tendency of CA1-PCs to fire at higher frequencies at the beginning of a depolarizing stimulus and then to accommodate. The analysis of “burstiness” of CA1-PCs in 20–23 month old PDAPP mice showed no difference between genotypes (Figures 5A–D); although, the IF profile for a 300 pA current injection was significantly different between PDAPP and WT controls, but due to the higher constant level of IF following accommodation (Two-way ANOVA: Source of variation: Genotype; DF = 1; *F* = 6.566, *p* = 0.011; Figure 5D), not to the burstiness (i.e., the first APs fired, before accommodation).

**Figure 6:**
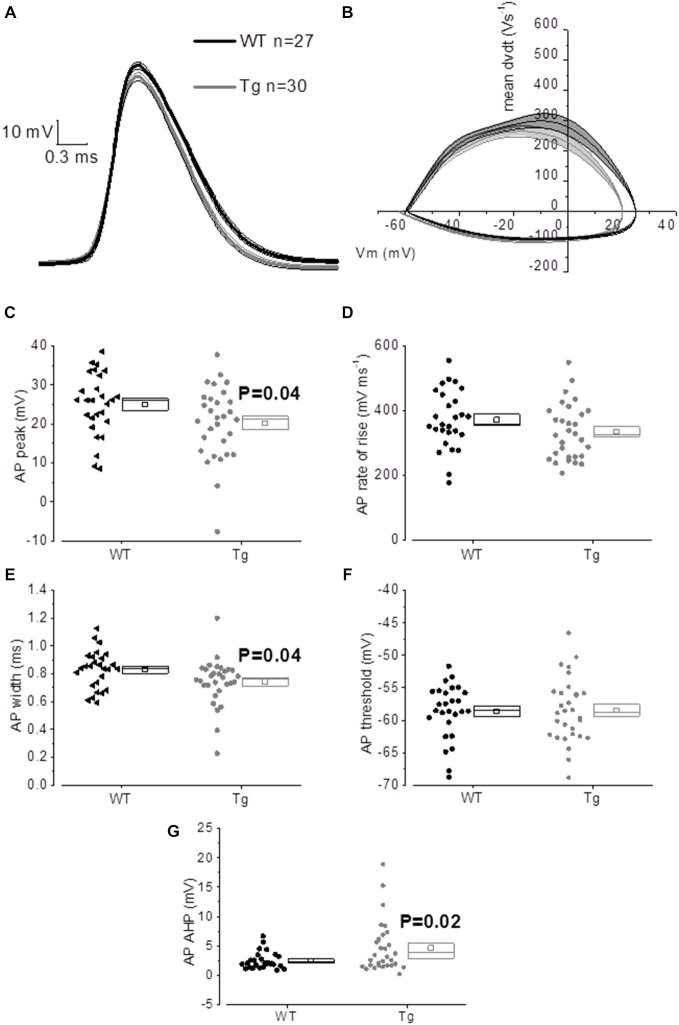
**20–23 month old PDAPP mice show an altered AP waveform. (A)** The averaged waveform, for each genotype, of the first AP elicited by the application of a 300 pA, 500 ms current injection: the thicker line represents the mean, which is delimited by its respective SEM (thinner line of the same color). **(B)** Phase-plot of the averaged AP waveforms for each genotype: the rate of change of the V_m_ during the AP is plotted against the V_m_. **(C–G)** Cell-to-cell analysis of the AP waveform properties. As already suggested by the averaged waveform and phase-plot, aged PDAPP mice showed a significantly more hyperpolarized zenith **(C)**, a thinner width **(E)** and a more hyperpolarized after-hyperpolarization (AHP) **(G)** but no difference in maximal rate of rise **(D)** and threshold **(F)**. See Table 3 for average ± SEM and *p* values.

The properties of the first AP fired in response to application of a 500 ms, +300 pA square current were studied. The average of the single AP waveform traces ± SEM in Figure 6A and of the rate of rise (RoR) plotted vs. V_m_ in Figure 6B indicate that genotype had an effect on AP properties. Cell-to-cell analysis of AP properties revealed that the AP zenith was significantly more hyperpolarized in PDAPP mice compared to littermate controls (Figure 6C; *p* = 0.04), as already observed in the time-course of the series of AP zeniths in response to +300 pA stimulations (Figure 4D). In addition, cells from PDAPP mice possessed significantly narrower APs (Figure 6E; *p* = 0.04), but no differences were seen neither in the maximum RoR of AP (Figure 6D; *p* = 0.13) nor in the AP threshold (Figure 6F; *p* = 0.88). The narrowing of the AP is consistent with previous observations in the same model at younger ages (Kerrigan et al., 2014) in PSAPP mice (Brown et al., 2011) and CRND8 mice (Wykes et al., 2012), while the reduced AP zenith is in accordance with the study on PSAPP mice (Brown et al., 2011). Finally, the AHP of the first AP evoked by a +300 pA current step was significantly bigger in PDAPP vs. WT controls (Figure 6G; *p* = 0.02): the increased AHP amplitude in PDAPP mice is a novel finding and can explain the lack of significantly increased firing rate observed here in comparison with the younger PDAPP mice (Kerrigan et al., 2014) and other models of amyloidopathy (see “Discussion” section for details).

**Table 3:** **Action potential properties of CA1 pyramidal neurons in hippocampal slices from 20 to 23 month old PDAPP mice and age-matched WT littermate controls**.

	WT *n* = 27		PDAPP *n* = 30	
Property	Average	SEM	Average	SEM	*p*
AP_peak (mV)	25.05	1.53	20.24	1.71	0.04
AP_width (ms)	0.83	0.03	0.74	0.03	0.04
AP_thres (mV)	−58.64	0.81	−58.46	0.90	0.88
AP_max_dvdt (Vs^−1^)	371.43	17.43	335.21	15.73	0.13
AP_AHP (mV)	2.46	0.28	4.62	0.80	0.02

**Figure 7:**
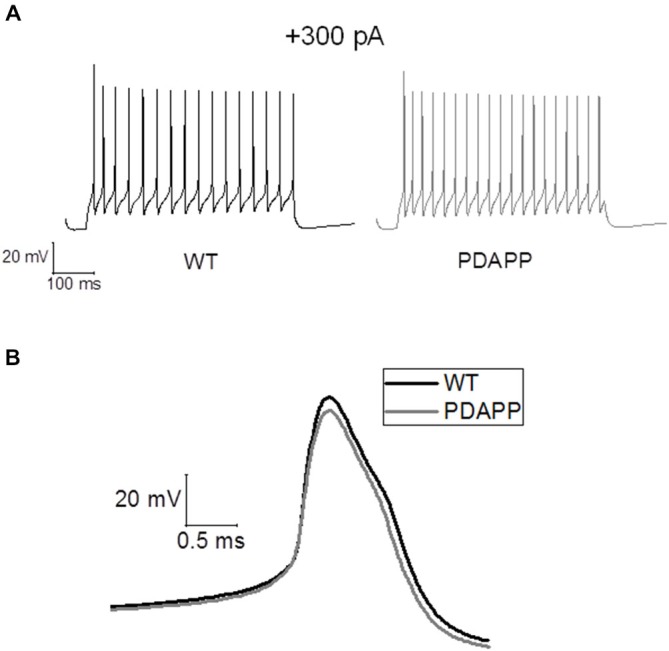
**Simulations of AP waveforms from 20 to 23 month old PDAPP mice and age-matched WT littermate controls based on a *in silico* model of a CA1/CA3 pyramidal neuron. (A)** AP firing model cell, following a 500 ms, 300 pA current step. The model produces 16 spikes in 500 ms in the case of WT parameter set and 20 spikes in the case of PDAPP. **(B)** Waveform of the first AP fired by the model cell in response to a 500 ms, 300 pA current step. The AP zenith and width are, respectively: WT: 25.41 mV and 0.84 ms—PDAPP: 20.21 mV and 0.73 ms.

For *in silico* modeling, in order to reproduce the behavior of an average (in terms of number of spikes) WT CA1 pyramidal neuron following the injection of a 500 ms, +300 pA square wave current (Figure 7A), we tuned the original model (Nowacki et al., 2011) parameters as follows: transient sodium conductance, g_NaT_ = 35.02 mS/cm^2^, g_NaP_ = 0.3 mS/cm^2^, g_Kdr_ = 13.8 mS/cm^2^, g_Km_ = 1.4 mS/cm^2^, g_CaH_ = 2.6 mS/cm^2^, g_CaT_ = 0.45 mS/cm^2^, g_Leak_ = 0.02 mS/cm^2^; the rest of the model parameters are the same as in Nowacki et al. (2011). We found that in order to reproduce (in the model) the exact change in the average values of the first AP zenith and width observed experimentally, it is sufficient to reduce the voltage-gated transient Na^+^ (to hyperpolarize the AP peak) and increase the voltage-gated K^+^ conductance (to narrow the AP width) as shown in Figure 7B: g_NaT_ = 23.04 mS/cm^2^, g_NaP_ = 0.5 mS/cm^2^, g_Kdr_ = 16.4 mS/cm^2^, g_Km_ = 1.2 mS/cm^2^, g_CaH_ = 2.6 mS/cm^2^, g_CaT_ = 0.45 mS/cm^2^, g_Leak_ = 0.02 mS/cm^2^. Thus our modeling directly supports the experimental observations and suggests that Na^+^ and K^+^ voltage-gated conductance biophysical property alterations might underlie the AP waveform changes observed in this transgenic mouse model of amyloidopathy.

**Figure 8:**
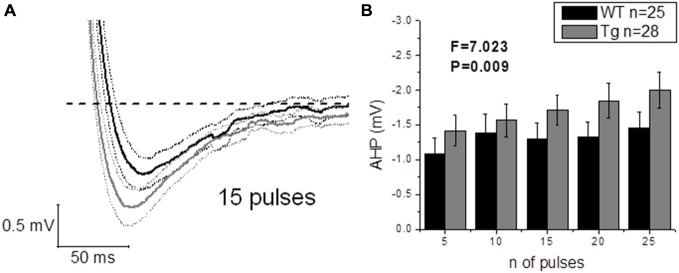
**20–23 month old PDAPP mice show an increased mAHP profile**. The injection of 50 Hz trains composed of 5–25 short (2 ms) and intense (2 nA) current steps resulted in the consequent evocation of single AP trains, followed by a mAHP, measured as the difference between the pre-train V_m_ (−80 mV) and the minimum reached in a time window of 500 ms from the end of the train. Genotype showed a significant effect in amplifying the mAHP in PDAPP mice vs. age-matched WT littermate controls. **(A)** Averaged traces ± SEM of mAHPs evoked by a 50 Hz, 15 pulses train. **(B)** Histogram showing the effect of genotype over mAHP.

The application of a short (2 ms) and intense (2 nA) square current pulse has been used to better isolate the effect of genotype on post-spike events, such as ADP and AHP: one single evoked AP did not produce different ADPs (WT: *n* = 22, 17.3 mV ± 1.5 mV vs. Tg: *n* = 24, 16.3 mV ± 1.0 mV; *p* = 0.6, unpaired Student’s *t*-test) or AHPs (WT: *n* = 25, −0.6 mV ± 0.1 mV vs. Tg: *n* = 29, −0.8 mV ± 0.1 mV; *p* = 0.3, unpaired Student’s *t*-test) between genotypes. In order to further investigate the AHP, trains of 5–10–15–20–25 2 ms/2 nA pulses were delivered at 50 Hz, to evoke trains of a defined number of APs, and the peak mAHP was measured (see “Materials and Methods” for details). Figure 8A shows the mean traces ± SEM for a mAHP evoked by 15 pulses delivered at 50 Hz. PDAPP mice showed a more hyperpolarized mAHP peak compared to WT littermate controls (Two-way ANOVA: Source of variation: Genotype; DF = 1; *F* = 7.023, *p* = 0.009; Figure 8B). This result is consistent with the AHP measured from the first AP fired in response to a +300 pA, 500 ms square pulse (Figure 6G) and what previously observed in 8 month old 5xFAD mice (Kaczorowski et al., 2011).

## Discussion

In this study we explored how various IE properties were altered in CA1-PCs in 20–23 month old PDAPP mice, an Aβ-overexpressing line created using transgenic expression of the *Indiana* mutation of APP. We achieved this by performing CC whole-cell electrophysiological recordings of visually targeted pyramidal neurons in the stratum pyramidale of the CA1 subfield of the hippocampus, in horizontal brain slices prepared from PDAPP and age-matched WT littermate controls. The RMP and other sub-threshold IE properties were investigated; the latter using standard electrophysiological protocols based on applied current injections. These experiments revealed no effect of genotype on most of the passive membrane properties explored, which included R_in_, SAG and resonance properties, as previously observed in Aβ-overexpressing transgenic mice (Brown et al., 2011; Wykes et al., 2012; Kerrigan et al., 2014; Kerrigan and Tamagnini, unpublished observations). In contrast Minkeviciene et al. (2009) showed that L2/3 neocortical pyramidal neurons and dentate granule cells from an early (3.5 mo) chronic model of amyloidopathy (APd9 Tg mice) have a more depolarized RMP, compared to their counterpart from age-matched WT controls. Our data are in agreement with a lack of change of RMP in CA1 pyramids of 3 other Aβ overproducing mouse lines (PSAPP, Tg2576, TAS-TPM) we have studied. This is interesting, because it underscores the concept that chronic exposure to Aβ species can have differential effects on the passive properties of different cell-types at different stages of the disease. Minkeviciene et al. (2009) result is supported by several studies testing the effects of acute exposure to high doses of Aβ toxic species (800 nM–10 μM), that showed a similar depolarizing and/or hyperpolarizing effect on the RMP of PC12 cells (Blanchard et al., 2002), basolateral amygdala neurons (Ashenafi et al., 2005) and CA3 pyramidal neurons (where R_in_ also increased; Nava-Mesa et al., 2013). These results tempt us to speculate that acute/early amyloidopathy might relatively affect RMP and R_in_; the consequent increased excitability this elicits, might then induce long term (chronic toxicity) compensatory changes in cell physiology, pushing to bring back RMP and R_in_ to physiological levels but altering AP waveform properties and cell firing. Interestingly enough, 20–23 month old PDAPP mice displayed a significantly lower capacitance compared to age-matched WT controls; such a reduction has not been previously observed in the same model, when tested at younger ages (Kerrigan et al., 2014) nor in other Aβ-overexpressing transgenic models of amyloidopathy (Brown et al., 2011; Wykes et al., 2012). Such a reduction of capacitance has not also been observed as a consequence of physiological aging. Thus, we can speculate that this novel observation, maybe due to reduced cell size, might be a direct consequence of the interaction between chronic exposure to toxic Aβ species and aging.

We also investigated suprathreshold firing properties. No difference was observed in terms of average firing properties even though PDAPP CA1-PCs displayed higher IF of firing after accommodation, when stimulated with strong (300 pA) current injections. This was unexpected, because at a younger age amyloidopathy has been consistently associated with an increased firing frequency both in PDAPP (Kerrigan et al., 2014) and other models of the disease (Brown et al., 2011; Wykes et al., 2012; Scala et al., 2015); in addition, in the present study, there appears to be a trend, although not significant, towards higher average frequencies of firing. The explanation for this observation may come from the analysis of AHP waveform properties. The AHP, both during a constant depolarizing square current injection (Figure 6G) and following trains of short, intense current injections (Figure 8) was larger in aged PDAPP mice compared to their controls. This was different from what we previously observed in this model at younger ages, where the mAHP was significantly smaller compared to age-matched WT controls (Kerrigan et al., unpublished observations) and in 1 month old WT slices pre-treated with Aβ (Tamagnini et al., 2015). However, this finding is consistent with previous work on 5xFAD mice which showed an age dependent increase of the AHP in transgenic mice compared to their controls (Kaczorowski et al., 2011). The increased AHP may explain why there is no difference in the average firing in aged PDAPP mice vs. WT controls, providing some evidence on how amyloidopathy and ageing can interact and perhaps contrast each other’s effects. Other interesting differences were observed in the AP waveform properties of aged PDAPP mice. Cell-to-cell analysis revealed that PDAPP CA1-PCs had narrower spikes and their AP zenith was more hyperpolarized. It appears that the narrowing of the AP width is a common feature of CA1-PCs in mouse models of amyloidopathy. Our group, along with others has observed these changes in PSAPP (Brown et al., 2011), younger PDAPP (Kerrigan et al., 2014), CRND8 mice (Wykes et al., 2012) and more recently in the TAS-TPM model as well (TK, FT, JB and AR, unpublished observations). This phenomenon has been ascribed to an increase of the voltage-gated K^+^ conductance in amyloidopathy: in Wykes et al. (2012) it has been showed that the narrowing of the AP width correlates with and it may causally be related to an increased expression of Kv3.1 channels. Such a mechanistic correlate is confirmed, in the present study, by the use of an *in silico* model (see “Results” section) based on the Hodgkin-Huxley CA1/CA3 pyramidal neuron model described in Nowacki et al. (2011): after we tuned the model to approximate the average AP waveform from the WT data population, an increase of voltage-gated K^+^ conductance was necessary to reduce the AP width to the PDAPP average value. Intriguingly, in the study by Scala et al. (2015) the opposite phenotype in CA1-PCs from 3xTg mice was observed. Even if the CA1-PCs studied in this latter research work showed higher firing rates compared to their controls, the APs were wider and the expression of Kv4.1 decreased: it is not easy to identify a reason for this discrepancy. The fact that the 3xTg model combines both amyloidopathy and tauopathy, however, suggests that the interaction between the two pathogenic pathways may result in phenotypes which are not as apparent in separate models of these two major pathological hallmarks of AD. We also previously observed a decrease of the AP zenith in 9–10 month old PSAPP mice (Brown et al., 2011), which are characterized by a double mutation, developing severe amyloidopathy at much earlier ages compared to PDAPPs (Elder et al., 2010). The interaction between PDAPP genotype and age could explain why it was possible to observe a reduction of the AP zenith in 20–23 month old PDAPP mice (as we previously reported for 9–10 month old PSAPP mice; Brown et al., 2011) but we could not see it in 8–9 month old PDAPP mice. In Brown et al. (2011) it has been shown that the hyperpolarized AP peak correlated with decreased maximal conductance of somatic voltage-gated Na^+^ currents in PSAPP vs. littermate controls: in the present study, the *in silico* model systematic investigation of voltage-gated conductances, suggests that the reduced AP peak observed in 20–23 mo PDAPP mice might be causally related to the decreased voltage-gated Na^+^ conductance.

In summary, considering the present observations, the modeling and the literature, it is reasonable to conclude that the AP width narrowing and AP peak hyperpolarization are likely to be specifically linked to an increased and decreased expression of voltage-gated K^+^ and Na^+^ channels, respectively, as a consequence of chronic exposure of CA1-PCs to toxic Aβ species.

To date there is a plethora of evidence demonstrating that amyloidopathy is clearly linked to aberrant epileptiform network activity in the hippocampus of both AD patients and transgenic animal models of the disease (Amatniek et al., 2006; Palop et al., 2007; Minkeviciene et al., 2009; Palop and Mucke, 2009, 2010; Born et al., 2014; Davis et al., 2014; Marcantoni et al., 2014). This is probably also partly due to the increased IE of CA1-PCs, as previously shown in both our and other laboratories (Brown et al., 2011; Wykes et al., 2012; Kerrigan et al., 2014; Scala et al., 2015). In direct contrast, physiological ageing has been associated with hypoexcitability in the same cell-types already discussed in WT control mice of the same genetic background (C57BL6) (Disterhoft and Oh, 2007; Randall et al., 2012). Such hyper- and hypo-excitability, in amyloidopathy and ageing respectively, thus, manifest themselves as either an increase or a decrease in the firing rate. In different models of amyloidopathy, the reduction of AP width has now been fairly consistently reported (Brown et al., 2011; Wykes et al., 2012; Kerrigan et al., 2014). This highlights the possibility that the narrowing of AP width, could lead to the observed increased excitability: the narrower is the AP the higher is the maximal firing rate a neuron can reach. Our findings illustrate various IE modifications in 20–23 month old PDAPP mice vs. their age-matched WT controls: some of the hyperexcitability features observed here can be correlated with those already observed in other models of amyloidopathy. These include reduced AP width and hyperpolarized AP zenith; however, no difference in the average firing rates was seen. The lack of difference in AP firing could be ascribed to the decreased cell capacitance observed, which may correspond to better short-circuiting of charge separation. It is also of interest to highlight the increase in the AHP in aged PDAPP mice: this phenotype was actually reversed in younger PDAPPs, which showed a smaller mAHP compared to WT controls (Kerrigan et al., unpublished observations). Such an age related increase in the AHP could therefore account for the lack of any change in the firing rate, even in presence of sharper and smaller spikes. In CA1-PCs, the mAHP relies on the activation of M-currents (I_M_) at depolarized potentials (i.e., −60 mV) and on the activation of I_h_-currents at more polarized potentials (i.e., −80 mV) (Gu et al., 2005); in this study we observed a bigger AHP during firing (that is to say: starting from a depolarized potential) consistently with a narrower AP width: both phenomena may causally relate to bigger I_M_ currents as a result of increased levels of voltage-gated K^+^ currents in PDAPP mice. On the other hand, we also observe a bigger mAHP starting from a hyperpolarized potential (i.e., −80 mV). Even if it is not possible to completely rule out a residual role for I_M_ in the generation of a mAHP at this voltage, such mAHP should mainly rely on I_h_, suggesting that this current should be up-regulated in PDAPP mice; if this was the case, however, we should also probably have observed an increase in sag, which we did not. For this reason, the change in the mAHP that we observed in older PDAPP mice is an interesting phenomenon that deserves further investigation, in order to better clarify its mechanistic correlates.

This study shows an interesting interaction between genotype and ageing in altering the IE of CA1-PCs in amyloidopathy. From these observations it is possible to conclude, when comparing with findings from the same model at younger tested ages, or other models of amyloidopathy and of physiological ageing, that:

1. the AP width is consistently reduced between models of amyloidopathy and across ages, presumably due to increased expression of voltage-gated K^+^ channels;
2. the AP zenith tends to get hyperpolarized depending on the severity of the amyloidopathy (and hence on the time needed to reach that degree of severity), presumably due to decreased expression of voltage-gated Na^+^ channels;
3. other properties, such as the firing rate, might depend on the interaction between normal ageing (which pushes towards hypoexcitability, maybe through the decreased capacitance and/or the increased AHP) and an Aβ-overexpressing genotype (which pushes towards hyperexcitability).

Although point (3) is a fascinating hypothesis, we cannot completely rule out the chance that some of these observations could be the result of some model specific effects: this possibility can only be explored by collecting more data from different Aβ-overexpressing transgenic mouse models at different age-points, which is what our lab has been strongly focusing in the last few years.

These results are of importance, as they highlight the delicate interaction between genotype and physiological ageing, which can differentially affect neuronal functioning at different stages of the disease progression. Such findings justify the immediate need to develop therapeutic strategies which consider, along the progression of the disease, the change in time of the therapeutic targets.

## Funding

Grant Sponsor: Medical Research Council (MRC); Grant number: G1100623.

## Conflict of Interest Statement

The authors declare that the research was conducted in the absence of any commercial or financial relationships that could be construed as a potential conflict of interest.
